# *Gli1^+^* Cells Exhibit Clonogenicity and Slow-Cycling Features at the Temporomandibular Joint (TMJ) Enthesis–Condyle Interface

**DOI:** 10.3390/ijms27073324

**Published:** 2026-04-07

**Authors:** Rafael Correia Cavalcante, Honghao Zhang, Peter X. Ma, Yuji Mishina

**Affiliations:** 1 Department of Biologic and Materials Sciences, The University of Michigan, Ann Arbor, MI 48109, USA; rafaelcc@umich.edu (R.C.C.);; 2 Department of Materials Sciences and Engineering, The University of Michigan, Ann Arbor, MI 48109, USA

**Keywords:** temporomandibular joint, temporomandibular joint disorders, clonogenic cell assays, progenitor cells, Ellis-van Creveld Syndrome, EVC2, mechanotrasduction

## Abstract

The temporomandibular joint (TMJ) relies on specialized progenitor cells for tissue maintenance and repair. We characterized TMJ-derived progenitor cells in mice and investigated the role of *Evc2*-mediated Hedgehog signaling. Progenitor cells from the anterior TMJ exhibited greater colony-forming capacity and an elongated morphology, while posterior cells were cuboidal, highlighting regional heterogeneity. TMJ-derived progenitors demonstrated multipotency, differentiating into osteogenic and chondrogenic lineages. *Gli1*-expressing, slow-cycling cells localized to the ligament attachment regions, initially accumulating there and not overlapping with specialized cells (Col1^+^ cells). Conditional *Evc2* disruption in *Gli1*-expressing cells paradoxically augmented expression of *Gli1* and mechanosensors (*Yap*, *Wwtr1*, *Piezo1*), and produced more confluent, rapidly expanding colonies. We hypothesize that these colonies are primarily composed of transit amplifying cells (TACs), which may proliferate robustly but face challenges in terminal differentiation. These results reveal critical roles for EVC2 and regional progenitor cell diversity in TMJ regenerative biology and suggest that targeting cell signaling and mechanical factors may inform novel strategies for TMJ disorder therapies.

## 1. Introduction

A fundamental aspect of study driving advances in tissue engineering is the expanding knowledge of tissue-specific stem cell biology [1,2]. Over recent decades, pivotal studies have established the potential of stem-cell based therapies, primarily through protocols involving stem cell in vitro expansion followed by transplantation [3]. However, these approaches encounter several significant obstacles. Engraftment efficiency is hindered by the distinct microenvironmental conditions present in different organs. Moreover, processes involving cell expansion and transplantation can lead to risks of transmissible infection. There is also a concern for tumorigenesis, largely attributed to suboptimal regulation of the cell cycle following engraftment. Lastly, immune-mediated rejection of transplanted cells remains a considerable challenge, limiting the long-term success of these therapies [3,4,5].

The temporomandibular joint (TMJ) is a complex anatomical structure comprising bones, cartilage, and associated musculature [6]. Anatomically, the TMJ connects the mandible to the midface, enabling it to withstand mechanical loads and facilitating a broad range of jaw movements. These dynamics are essential for fundamental activities such as speaking, breathing, and mastication [6,7]. Temporomandibular joint disorders (TMD) refer to a group of conditions that collectively result in pain, dysfunction, and disability, affecting approximately 20–25% of the global population [6,8,9]. Although the precise cellular origin of the TMJ remains incompletely defined, current evidence indicates that both neural crest cells and cells from the mandibular bone periosteum contribute to its development [10]. During embryogenesis, cellular condensation within the TMJ initiates the formation of a cartilage anlage, which is subsequently resorbed by chondroclasts and replaced by mineralized bone [11]. Notably, in contrast to the growth plate cartilage found in long bones, which is entirely resorbed upon skeletal maturation, the TMJ cartilage persists into adulthood, serving as durable articular cartilage within the joint [12]. This persistent mandibular condylar cartilage is uniquely capable of adaptive remodeling in response to functional and biomechanical stimuli, such as external force or mandibular repositioning [13]. Emerging evidence from lineage tracing and molecular studies further demonstrates that, unlike the classic model of chondrocyte apoptosis, TMJ hypertrophic chondrocytes can directly transform into bone cells, actively contributing to endochondral ossification and joint maintenance [14].

The enthesis of the TMJ is the specialized anatomical region where connective tissues, such as the joint capsule and ligaments, attach to osseous structures like the mandibular condyle and temporal bone [15,16]. This interface is histologically complex and consists of several transition zones: a dense fibrous zone providing tensile strength, unmineralized and mineralized fibrocartilaginous layers that accommodate compressive and shear forces, and the underlying subchondral bone which anchors the soft tissues [17,18]. Functionally, the TMJ enthesis is essential for transmitting and dissipating mechanical loads generated during jaw movement, thereby protecting joint integrity [15,16,17,18]. Emerging evidence from studies on the knee joint suggests that the enthesis serves as a niche for resident progenitor cell populations, such as *Gli1^+^* cells [19,20]. These cells have the potential to contribute to tissue maintenance, repair, and adaptation in response to mechanical stress within the joint [20,21]. Recently, our group demonstrated that neural crest-specific disruption of *Evc2*, which is known as a modulator of Hedgehog (HH) signaling, combined with altered jaw loading, induced structural abnormalities in the TMJ and significantly upregulated the expression of genes associated with osteoarthritis-related markers [22].

In this study, we provide the first characterization of a progenitor cell population that is predominantly localized within the TMJ enthesis and exhibits robust colony-forming potential, as demonstrated by colony-forming unit fibroblast (CFU-F) assays. These cells are *Gli1^+^* and display distinct progenitor cell properties, including multilineage differentiation capacity. To investigate the regulatory mechanisms underlying their function, we introduced a conditional knockout (cKO) mutation of *Evc2* specifically in *Gli1*-expressing cells expecting that this would downregulate the HH signaling. This targeted genetic manipulation resulted in enhanced engraftment and a significant increase in CFU-F colony density, suggesting that HH signaling through EVC2 plays a critical role in supporting the proliferation and engraftment of TMJ enthesis-derived progenitor cell populations.

## 2. Results

### 2.1. TMJ Contains CFU-F Exhibiting Multi-Lineage Differentiation Potential

**Figure 1:**
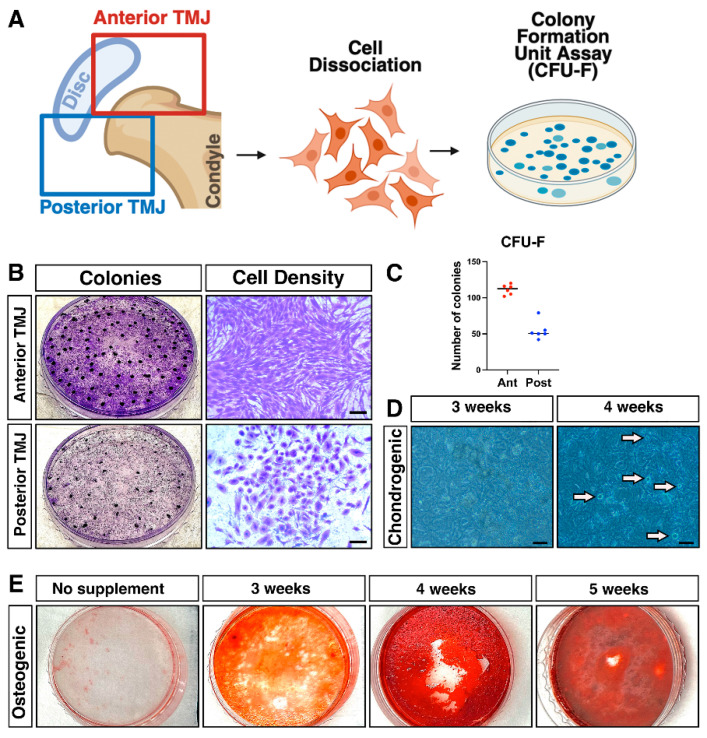
TMJ Contains CFU-F with Multi-Lineage Differentiation Potential. (**A**) Schematic of TMJ cell isolation and culture. TMJ primary cells were harvested from 8–12-week-old mice by dissecting a ~2.0 mm wide section including the condyle cartilage layer, the ligament attaching sites, and the TMJ disc, followed by enzymatic dissociation and filtration. (**B**) CFU-F assay and identification. Isolated cells were plated at 1 × 10^5^ cells per dish and cultured for 15 days. Colony-forming units were defined as clusters of >60 cells after crystal violet staining. (**C**) Quantification of CFU-F colonies demonstrates robust colony-forming potential in TMJ-derived cells. (**D**) Chondrogenic differentiation of TMJ-derived colonies. Following CFU-F culture, colonies were subjected to chondrogenic induction using the StemPro™ Chondrogenesis Differentiation Kit and evaluated by Alcian Blue staining for sulfated glycosaminoglycans at 3 and 4 weeks. Arrows indicate micro-mass stain. (**E**) Osteogenic differentiation of TMJ-derived colonies. Colonies were cultured in osteogenic differentiation medium for 3, 4, or 5 weeks and assessed by Alizarin Red S staining for calcium deposition. Bar = 50 μm (images in panels (**B**,**D**)).

Our first objective was to characterize progenitor cell populations within the TMJ by assessing their colony-forming potential, multipotency, and anatomical localization. Cells isolated from mouse TMJs were cultured at basal medium supplemented with 2-mercaptoethanol to evaluate their ability to form colonies. The TMJ was anatomically divided into anterior and posterior regions, from which cells were subsequently isolated for further analysis (Figure 1A). Under these conditions, the TMJ-derived progenitor cells exhibited robust clonogenic potential, as evidenced by the formation of distinct, well-developed colonies after plating. CFU-F assays revealed that cells isolated from the anterior region of the TMJ exhibited a significantly greater capacity to form colonies compared to those derived from the posterior region (Figure 1B,C). In addition to their enhanced clonogenic potential, cells from the anterior region displayed a distinct elongated morphology, while cells isolated from the posterior region primarily exhibited a cuboidal shape (Figure 1B). These findings indicate regional differences not only in the abundance and colony-forming ability of TMJ progenitor cells but also in their cellular morphology, which may reflect functional heterogeneity within the joint (Figure 1B).

To further investigate their multipotency, the colonies were subjected to specific induction protocols designed to promote differentiation into chondrogenic (cartilage-forming) and osteogenic (bone-forming) lineages. Following these treatments, the primary cells displayed clear evidence of differentiation, successfully adopting characteristics typical of both bone (stained using Alizarin Red S) and cartilage cells (stained using Alcian Blue) (Figure 1D,E). These results demonstrate that TMJ-derived progenitor cells possess strong colony-forming capabilities and the versatility to differentiate into multiple cell types.

### 2.2. Evc2 Disruption in Gli1-Expressing Cells Paradoxically Enhances Gli1 and Mechanosensor Gene Expression in TMJ-Derived Cells

**Figure 2:**
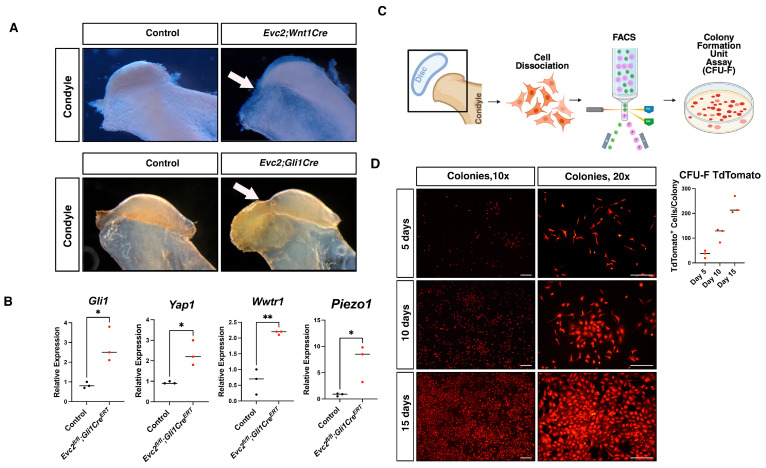
*Evc2* Disruption in *Gli1*-Expressing Cells Enhances *Gli1* and Mechanosensor Gene Expression in TMJ-Derived Cells. (**A**) Macroscopic images of TMJ from control, *Evc2^fl/fl^*; *Wnt1Cre*, and *Evc2^fl/fl^*; *Gli1Cre^ERT^* mice, illustrating anatomical differences (white arrows). (**B**) Quantitative RT-PCR analysis of TMJ tissues shows augmented expression of *Gli1* and mechanosensor genes (*Yap1*, *Wwtr1*, and *Piezo1*) in *Evc2* mutant mice when compared to controls. (**C**) Schematic of experimental workflow for assessing clonogenic potential of *Gli1*-expressing TMJ cells. *Gli1Cre^ERT^*; *R26R^TdTomato^* reporter mice were used for FACS sorting and isolation of TdTomato-positive (*Gli1*-expressing) cells, which were then cultured at low density. (**D**) Colony formation from sorted TdTomato-positive cells was assessed at 5, 10, and 15 days post-plating. All colonies consisted entirely of TdTomato-positive cells, confirming that colony-forming activity originated from *Gli1*-expressing progenitor cells. *, *p* < 0.05, **, *p* < 0.01. Bar = 200 μm (upper, middle, and bottom images from the left column in panel (**D**)) and 50 μm (upper, middle, and bottom images from the right column in panel (**D**)).

Building on the identification of TMJ cells with progenitor-like features, we next investigated molecular mechanisms regulating their behavior, focusing on HH signaling via EVC2 mutations. As we previously reported, neural crest-specific disruption of *Evc2* develops TMJ-OA like phenotypes that is significantly and quickly restored by soft diet treatment [22]. Together with the fact that EVC2 is a positive modulator for HH signaling [23,24,25,26], we hypothesized that EVC2 plays a critical role in presumptive TMJ progenitor cells by regulating HH signaling and mechanosensory pathways. Thus, we utilized *Evc2^fl/fl^*; *Gli1Cre^ERT^* mice to conditionally delete *Evc2* in *Gli1*-expressing cells. The mutant mice showed similar TMJ-OA phenotypes found in neural crest-specific disruption of *Evc2* (Figure 2A). The mutant condyles were characterized by degenerative changes in the joint’s articular cartilage and subchondral bone showing focal cartilage erosion, thinning, and fibrillation, with loss of proteoglycan staining and surface irregularities. The underlying bone displayed sclerosis, subchondral bone thickening, and the formation of osteophytes (bony outgrowths). In contrast, control condyles retained a smoother cartilage surface with more uniform proteoglycan distribution and normal subchondral bone architecture. This suggests that EVC2 function in *Gli1*-expressing cells is responsible for TMJ-OA phenotype. Surprisingly, TMJ-derived cells from these mice exhibited a marked upregulation in *Gli1* mRNA levels, contrary to the expected downregulation (Figure 2B, Supplementary Table S1). In addition, gene expression analysis revealed that mechanosensory genes, including *Yap*, *Wwtr1* (*Taz*), and *Piezo1*, were also significantly upregulated in TMJ cells lacking *Evc2* (Supplementary Table S1). This suggests that loss of *Evc2* in TMJ-*Gli1*-expressing cells not only fails to suppress *Gli1* expression but promotes its upregulation alongside activation of mechanosensory pathways. This paradoxical response highlights a complex regulatory relationship between EVC2 and HH signaling within the TMJ microenvironment.

### 2.3. Colony-Forming Capacity of Gli1-Expressing Cells

Given the observed increase in HH and mechanosensory signaling, we sought to directly assess the clonogenic potential of *Gli1*-expressing cells in the TMJ and therefore we isolated TMJ-derived cells from *GliCre^ERT^*; *R26R^TdTomato^* reporter mice. FACS sorting was used to select and purify TdTomato-positive cells, ensuring the enrichment of cells specifically expressing *Gli1*. The sorted cells were then plated in 35 mm culture dishes at low density (Figure 2C). After attachment to the plate surface, colony formation was monitored at regular intervals, specifically at 5, 10, and 15 days post-plating. Over time, distinct colonies developed from the TdTomato-positive cells. At the conclusion of the experiment, all observed colonies were confirmed to be TdTomato-positive, indicating that the colony-forming activity was derived robustly from *Gli1*-expressing progenitor cells (Figure 2D). This demonstrates that *Gli1*-expressing cells within the TMJ population are capable of colony formation, suggesting their possible role as progenitors with regenerative potential.

### 2.4. Enhanced Colony-Forming Capacity in Evc2 Conditional Knockout TMJ Gli1-Expressing Cells

**Figure 3:**
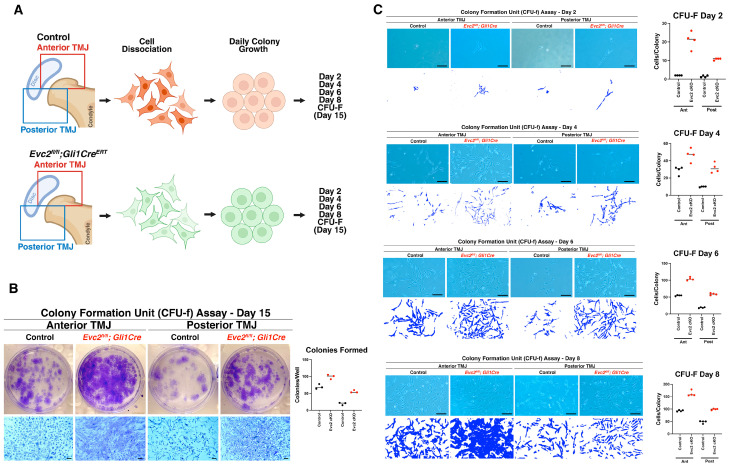
Enhanced Colony-Forming Capacity in *Evc2* Conditional Knockout TMJ *Gli1*-Expressing Cells. (**A**) Schematic of experimental workflow showing isolation of TMJ-derived cells from control (*Evc2^fl/+^*; *Gli1Cre^ERT^*) and *Evc2* cKO (*Evc2^fl/fl^*; *Gli1Cre^ERT^*) mice, with anatomical division of the TMJ into anterior and posterior regions. CFU-F from control and *Evc2*-disrupted cells were stained with crystal violet 15 days after initial incubation. Sequential observations of colony formation and expansion in TMJ-derived cell cultures from control and *Evc2* cKO mice were also recorded at days 2, 4, 6, and 8. (**B**) Quantitative CFU-F assay results demonstrating that Evc2-disrupted *Gli1*-expressing progenitor cells exhibit significantly greater colony-forming potential compared to controls after 15 days in culture. (**C**) Sequential observations of colony formation and expansion in TMJ-derived cell cultures from control and Evc2 cKO mice at days 2, 4, 6, and 8. *Evc2*-disrupted *Gli1*-expressing progenitor cells showed accelerated adhesion, engraftment, and early confluency (days 6–8), in contrast to the delayed confluency observed in control cultures. Bar = 50 μm (images in (**B**,**C**)).

To determine the functional impact of *Evc2* loss on progenitor cells dynamics across TMJ regions, we isolated cells from both control (*Evc2^fl/+^*; *Gli1Cre^ERT^*) and *Evc2* cKO (*Evc2^fl/fl^*; *Gli1Cre^ERT^*) mice, analyzing colony formation and expansion. The TMJ was anatomically divided into anterior and posterior regions, from which cells were subsequently isolated for the experiments (Figure 3A). Using the CFU-F assay, we assessed the clonogenic potential of these populations after 15 days in culture. Remarkably, cells isolated from *Evc2* cKO mice exhibited a significantly greater ability to form colonies compared to their control counterparts (Figure 3B). This unexpected finding aligns with our earlier gene expression data from TMJ tissues and led us to hypothesize that *Evc2*-deficient *Gli1*-expressing cells may preferentially expand as transit amplifying cells (TACs). TACs are a subset of progenitor cells characterized by their rapid proliferation and ability to transiently amplify cell numbers before differentiating into mature tissue-specific cells [27,28,29]. While TACs contribute to tissue regeneration, their accumulation may also indicate an altered balance between proliferation and differentiation in the absence of *Evc2*. These results suggest that *Evc2* disruption in TMJ *Gli1*-expressing cells augments colony formation, driving the expansion of a TA cell population. The accumulation of TACs may impact tissue homeostasis and repair dynamics within the TMJ, which could contribute to the TMJ-OA phenotypes observed in *Evc2* mutant mice.

Next, we performed daily observations of colony formation in cultures established isolating TMJ-derived cells from both control and *Evc2* cKO mice. Throughout the assessment period, *Evc2* cKO cells consistently demonstrated a superior capacity to adhere to and engraft onto the culture dishes. These cells quickly expanded into sizeable colonies at a rate that greatly exceeded that of the control group (Figure 3C). A key observation was that *Evc2*-deficient cells achieved confluency, much earlier than the controls, typically between days 6 and 8 following initial plating (Figure 3C, fourth panel row). In comparison, the control cells required a longer duration to reach confluency, signifying a distinct difference in proliferative speed and expansion capability. This pronounced increase in engraftment efficiency and rapid colony amplification in *Evc2* cKO TMJ-derived cells further highlights the critical role of EVC2 in maintaining the balance between progenitor cell proliferation and differentiation. The enhanced growth kinetics observed in the *Evc2*-deficient cells suggest that EVC2 serves as a modulator to restrain excessive cell amplification, thereby preserving tissue architecture and proper cell lineage commitment.

Interestingly, while *Evc2*-deficient TMJ progenitor cells demonstrate a higher colony-forming capacity, they may face challenges in differentiating into specialized, mature cell types. Our group’s recent publication supports this observation, when *Evc2* cKO mice are maintained on a regular diet, they exhibit significantly more structural defects in the mandibular condyle, accompanied by elevated expression of inflammatory markers in TMJ tissue [22]. These findings suggest that the absence of EVC2 not only promotes the proliferation of TACs but may also impair their ability to complete differentiation and tissue maturation, resulting in compromised joint structure and increased inflammation. This phenotype can be mitigated by dietary modification. When *Evc2* cKO mice are provided with a soft diet, the structural defects in the mandibular condyle are significantly rescued [22]. This highlights the impact of mechanical loading and environmental factors on TMJ biology, as well as the potential for dietary interventions to ameliorate the negative consequences of disrupted signaling pathways.

Biologically, these findings carry important implications. The augmented proliferation in the absence of EVC2 could potentially lead to an altered cellular composition within the TMJ, with possible effects on tissue regeneration, repair, and long-term joint integrity. While increased expansion might be beneficial for initial regenerative responses, unchecked proliferation of TACs could also disrupt normal differentiation pathways and contribute to pathological changes.

### 2.5. Localization of Slow-Cycling Cells in the TMJ Enthesis Identified by H2B-GFP Retention

**Figure 4:**
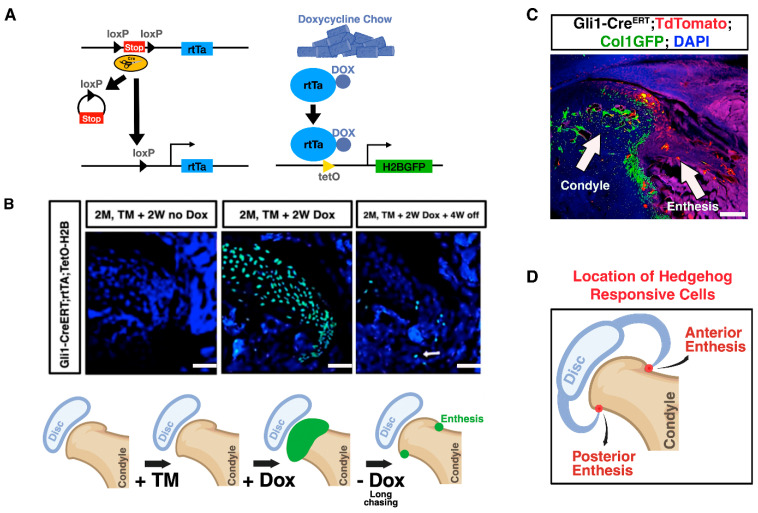
Localization of Slow-Cycling Cells in the TMJ Enthesis Identified by H2B-GFP Retention. (**A**) Schematic illustrating the use of *Gli1Cre^ERT^*; *rtTA*; *TetO-H2B-GFP* reporter mice for identifying slow-cycling cells within the TMJ, based on retention of the H2B-GFP label. Tamoxifen administration activates Cre^ERT^, which removes a stop codon, allowing for the transcription of the reverse tetracycline transactivator (rtTA) in *Gli1*-expressing cells. When the animals are subsequently fed doxycycline (Dox) chow, Dox binds to rtTA, enabling it to activate the TetO promoter and induce expression of the H2B-GFP fusion protein. (**B**) Following tamoxifen administration and Dox chow feeding, GFP expression was widespread across the TMJ articular surface, labeling actively cycling cells (green). After withdrawal of Dox for a 4-week chase period, GFP-retaining cells, indicating slow-cycling populations, became restricted to the enthesis region. (**C**) 24 h post-tamoxifen injection, analysis of *Gli1Cre^ERT^*; *R26R^TdTomato^*; Col1-GFP mice revealed TdTomato-positive cells (red) within the TMJ enthesis that did not overlap with specialized Col1-GFP-positive cells (green). (**D**) Schematic summarizing the TMJ enthesis as a niche harboring slow-cycling, Hedgehog (HH)-responsive stem/progenitor cell populations. Bar = 50 μm (left, middle, and right images in (**B**)) and 200 μm (image in (**C**)).

Recognizing that stem cell function often correlates with slow cell cycling (quiescent cells), we aimed to determine the spatial distribution and cycling kinetics of these cells. Specifically, we sought to identify quiescent, *Gli1*-expressing cells in the TMJ to better understand the localization of the progenitor cell niche and its potential role in tissue maintenance and regeneration. To investigate the spatial distribution of slow-cycling cells within the TMJ, we utilized *Gli1Cre^ERT^*; *rtTA*; *TetO-H2B-GFP* reporter mice. This system enables the identification of slow-dividing cells through retention of the histone H2B-GFP label (Figure 4A). Following TM administration and supplementing the mice’s diet with doxycycline (Dox) chow, robust GFP expression was observed throughout the articular surface of the TMJ, indicating successful labeling of actively cycling cells. Subsequently, Dox was withdrawn from the diet to initiate a “chase” period. Over time, as rapidly cycling cells lost the GFP label due to dilution, only slow-cycling cells retained the GFP signal. After a prolonged chase period (4 weeks), these GFP-retaining cells were predominantly localized within the enthesis region of the TMJ (Figure 4B). In addition, using *Gli1CreERT*; *R26RTdTomato*; *Col1-GFP* mice, we observed TdTomato-positive cells in the TMJ enthesis 24 h after tamoxifen injection. These TdTomato-positive cells did not overlap with the specialized Col1-GFP-positive cells (Figure 4C). Demonstrating that the enthesis of the TMJ serves as a niche for slow cycling, some of the HH responsive cells are likely stem/progenitor cell populations (Figure 4D).

### 2.6. Condyle Cartilage (CC) Cells Are Non-Clonogenic and Ligament-Derived Cells Form Less Robust Colonies Without CC Cells, Indicating Interdependency Between TMJ Progenitor Populations

**Figure 5:**
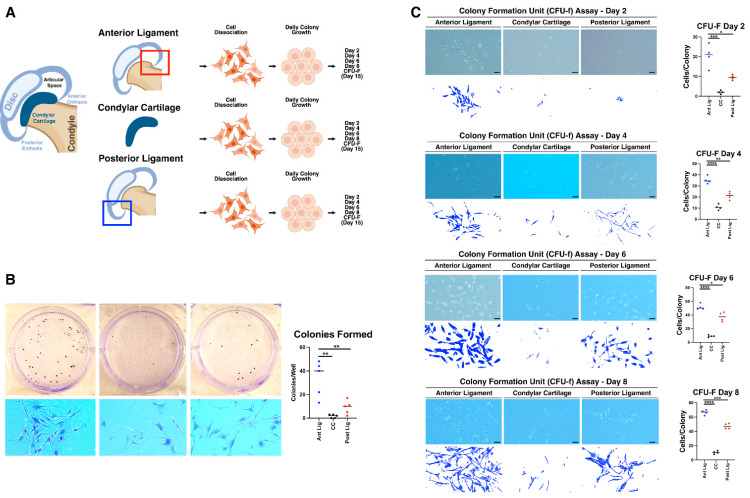
(**A**) Schematic of the experimental workflow showing the isolation of TMJ-derived cells from three compartments in control mice: anterior ligament (ant lig, red box), condyle cartilage (CC), and posterior ligament (post lig, blue box). CFU-F assays were stained with crystal violet 15 days post-incubation. Sequential observations of colony formation and expansion were recorded at days 2, 4, 6, and 8. (**B**) Quantitative CFU-F assay results demonstrating that cells from the anterior and posterior ligaments readily formed colonies, in contrast to cells from the CC, which showed minimal colony formation. (**C**) Sequential images of colony formation in cultures from anterior ligament, CC, and posterior ligament at days 2, 4, 6, and 8. Cells from anterior and posterior ligaments exhibited adhesion, engraftment, and confluency by day 8, whereas cells from CC showed few colonies and limited expansion. *, *p* < 0.05, **, *p* < 0.01, ***, *p* < 0.001, ****, *p* < 0.0001. Bar = 50 μm (images in panels (**B**,**C**)).

To confirm the anatomical location of clonogenic cells within the TMJ and assess possible interdependency, we dissected the joint from 2-month-old wild-type mice into anterior ligament, CC, and posterior ligament components (Figure 5A). CFU-F assays revealed that cells from the anterior ligament consistently formed colonies, while cells from the CC showed almost no colony formation (Figure 5B). Day-by-day evaluation indicated that CC-derived cells were able to attach to the culture surface but failed to develop colonies over time, showcasing their lack of clonogenic potential (Figure 5C). This demonstrates that clonogenic capacity is primarily restricted to the ligament regions, reinforcing the anatomical specificity of TMJ progenitor niches. Interestingly, when comparing the colony density, we found that cells isolated from the anterior and posterior ligaments alone produced less robust colonies (Figure 5B) than cultures combining both ligament and CC cells (Figure 3B). This finding suggests a possible interdependent relationship between ligament and cartilage cells in TMJ CFU formation, where the presence of both cell types may enhance the proliferative capacity or viability of the colonies.

## 3. Discussion

In human TMJ anatomy, differential loading patterns are well-recognized, with anterior and posterior joint regions experiencing unique mechanical stresses during jaw function. Our data reveal that TMJ ligament attachment sites, previously unappreciated anatomical regions, are a distinct niche for *Gli1*-expressing progenitor-like cells with strong colony-forming and multipotent differentiation abilities. The regional heterogeneity observed in cell morphology and CFU numbers likely reflects adaptation to local biomechanical forces, tissue organization, and microenvironmental cues relevant to joint function and disease. Recent research highlights that tissue stiffness varies spatially and temporally across the jaw and TMJ, and such heterogeneity may influence stem/progenitor cell behavior [30,31]. Furthermore, mechanical stiffness is increasingly recognized as a regulator of cell fate and metabolism, and biomimetic scaffolds are now used to model these properties in vitro [22].

The localization of *Gli1*-expressing slow-cycling cells within the TMJ ligament-attaching sites highlights its importance not only as a structural anchor but also as a key niche supporting the cellular dynamics required for joint maintenance. This notion well aligns with our recent finding that alteration of mechanical loading by soft diet treatment significantly restores structural abnormalities caused by *Evc2* cKO mice [22]. Our findings expand on a growing body of literature dedicated to identifying unique progenitor cell populations within the TMJ. To date, most studies have focused on progenitor cell pools located in the mandibular condyle, such as fibrocartilage stem cells (FCSCs) in the superficial zone, marked by alpha-SMA (a-SMACreERT2; Ai9) expression and stem cell features [32]. Additional research has highlighted Scleraxis (Scx)-lineage cells in the fibrous layer of the condyle, which can differentiate into prechondroblasts [33], and animal studies using Gli1 reporters (*Gli1Cre^ERT2^*; *TdTomato*) have identified positive cells within the condyle superficial zone [34,35]. Furthermore, *FSPI-Cre*; *mTmG* models found stem cells in the condyle’s superficial layer [30], and Lgr5 (*Lgr5EGFPcre^+-^*; *ROSA/TdTomato*) expression has been linked to the regulation of chondrocyte identity and the maintenance of chondroprogenitors in the superficial layer [36]. Unlike the articular surface, which has long been studied for its progenitor cell populations and regenerative capacity, the ligament sites integrate biomechanical function with cellular stewardship, serving both as a physical anchor and a dynamic reservoir for tissue renewal. The existence of *Gli1*-expressing slow-cycling cells, alongside a structurally demanding environment, points to a sophisticated interplay of molecular signals, lineage hierarchies, and adaptive responses unique to the joint ligament. This complexity elevates the TMJ ligament attaching sites from a mere connective interface to a multifaceted regulator of joint health.

Building on the identification of the ligament attaching sites as a central niche for TMJ progenitor cells, our exploration into the molecular regulation of these populations reveals further layers of complexity, particularly regarding HH signaling and *Gli1^+^* cell dynamics [34,35]. Notably, the absence of EVC2 demonstrated that perturbations in key signaling pathways can dramatically alter progenitor cell behavior [23,24,25]. The conditional disruption of *Evc2* in *Gli1*-expressing cells resulted in a paradoxical upregulation of *Gli1* and mechanosensory genes such as *Yap*, *Wwtr1*, and *Piezo1* in TMJ tissues. While EVC2 has been classically understood as a positive regulator of HH signaling in other tissues, including growth plate chondrocytes [26,37], its absence in the TMJ did not diminish pathway activity as expected; instead, loss of EVC2 amplified *Gli1^+^* and mechanotransductive signals, suggesting a regulatory role within the TMJ microenvironment. Recent studies demonstrate direct crosstalk between Hedgehog and mechanosensing pathways such as YAP/TAZ (Hippo); for instance, YAP activation can promote HH signaling and inhibit neuronal differentiation, with Shh signaling acting downstream of YAP [38,39]. Our findings highlight that *Evc2* disruption in the TMJ upregulates mechanosensory genes, and that soft diet can partially rescue these phenotypes [22]. Whether altered mechanosensing drives progenitor expansion or follows increased proliferation is still unclear, but developmental studies show mechanical stress activates both YAP and HH pathways [39], and similar mechanisms may operate in the TMJ, where expanding transit amplifying cells can dynamically remodel tissue architecture. While the paradoxical upregulation of Gli1 and mechanosensory genes following *Evc2* deletion is a robust and reproducible observation, its mechanistic basis remains unresolved. Our current data are correlative and highlight the need for targeted experiments, such as pathway inhibition, genetic rescue approaches, or cell signaling reporter assays, to directly dissect the underlying regulatory interactions. Future studies will be essential to distinguish among the proposed mechanisms and fully explain this regulatory phenomenon.

**Figure 6:**
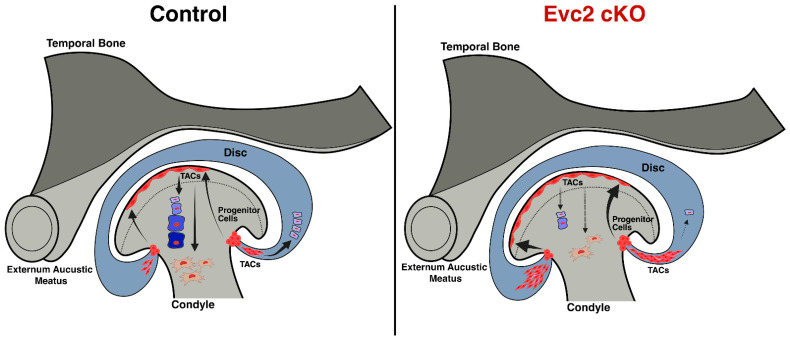
Schematic Model of Progenitor Cell Dynamics in the TMJ Enthesis and the Impact of Evc2 Disruption. A hypothetical model illustrating the location of progenitor cells in the TMJ enthesis. In normal conditions, these cells expand toward different anatomical regions of the joint, including the articular surface and disc, differentiating into TACs and subsequently specialized cartilage and bone cells. *Evc2* disruption accelerates proliferative activity, disrupting the balance between progenitor cell proliferation and differentiation. Such dysregulation may promote pathological tissue remodeling and chronic inflammation, highlighting the pivotal role of EVC2 in preserving TMJ homeostasis and protecting against disease processes arising from aberrant progenitor cell dynamics.

This altered signaling landscape shifted progenitor cell dynamics toward rapid proliferation, as observed by the robust in vitro expansion of colonies, populations we hypothesize to be predominantly composed of TACs (Figure 6) [27,40]. In the present study, we observed that unchecked proliferation in the absence of EVC2 dysregulates normal differentiation processes, compromising the joint’s long-term regenerative capacity. This imbalance threatens tissue integrity and has been reported to contribute to pathological outcomes in other tissues, such as fibrosis [41], osteoarthritis [42], or abnormal growth [43]. Our group previously demonstrated that loss of *Evc2* in dental mesenchymal stem cells within the cervical loop of mouse incisors leads to an upregulation in the proportion of TACs, resulting in impaired tooth development and enamel hypoplasia [44]. Our results in the TMJ parallel findings in other tissues [45,46], suggesting that EVC2-driven expansion of TAC populations may be a common regulatory mechanism [44]. The enrichment of TACs in *Evc2*-deficient colonies is inferred from their rapid proliferation, colony morphology, and engraftment speed, traits consistent with TACs, which lack specific molecular markers [45,46]. Our interpretation that *Evc2* disruption promotes TAC expansion is speculative and based on colony phenotypes such as growth kinetics and morphology. While these characters are consistent with TAC features described in other tissues [45,46], distinguishing TACs from long-term stem cells requires more rigorous methods like lineage tracing, single cell analysis, or functional assays, which are beyond the scope of this study. Therefore, our conclusions regarding progenitor hierarchy and lineage behavior should be considered preliminary and foundational for future mechanistic studies.

In addition, investigation into the engraftment and growth dynamics of *Gli1*-expressing progenitor cells revealed that *Evc2* cKO TMJ-isolated cells markedly enhance both colony formation and expansion. In vitro, *Evc2* cKO cells exhibited superior adhesion and rapid proliferation, reaching confluency between days 6 and 8, significantly earlier than their control counterparts. The accelerated proliferative activity showcases the EVC2’s pivotal role in maintaining the delicate balance between progenitor cell proliferation and differentiation. While our in vitro experiments provide valuable insight into colony-forming potential and progenitor cell dynamics, direct in vivo validation of their contributions to tissue maintenance, regeneration, or pathology is lacking. Thus, our conclusions about functional relevance and disease association should be regarded as hypotheses, and future studies with in vivo lineage tracing and functional assays will be essential to confirm these roles.

Expanding on the concept of interdependency within stem cell compartments, recent work in hair follicles has shown that TACs orchestrate stem cell activity and tissue regeneration through niche signaling [45]. Drawing parallels, our findings show that colonies are less robust when ligament-derived cells are cultured alone but more viable with condyle cartilage cells; these findings suggest that TMJ ligaments and the condyle cartilage may also collaborate in maintenance or regeneration. Our findings contribute to broader principles of joint progenitor cell regulation beyond the TMJ. The demonstration of Evc2-mediated crosstalk between Hedgehog signaling and mechanosensory pathways, as well as the interdependent relationship between ligament and cartilage-derived progenitor populations, underscores a regulatory logic not strictly tied to anatomical localization. These results suggest that niche signals and compartmental cooperation are essential for maintaining progenitor pool balance and regenerative capacity across joint tissues. By showing how somatic niche factors can reshape cellular hierarchies, driving transit amplifying cell expansion and modulating differentiation, our study establishes TMJ as a model system for investigating joint-wide mechanisms of progenitor regulation. This conceptual framework extends to other synovial joints, where integration of molecular, mechanical, and microenvironmental cues likely governs stem cell function and tissue homeostasis. Nevertheless, further investigation is necessary to clarify the roles and interactions among TMJ stem and progenitor populations.

Clinically, our findings outline potential therapeutic avenues: targeting EVC2, HH signaling, or the mechanical environment could fuel TMJ repair, but approaches must be calibrated to avoid excessive expansion of progenitor cells and attendant risks of pathology. Ultimately, restoring the balance between cell proliferation and differentiation, possibly through combined molecular and mechanical interventions, may optimize healing outcomes for TMJ injury and degeneration.

## 4. Materials and Methods

### 4.1. Mouse Genetic Generation

The study ensured that animal usage was ethically and humanely performed by adhering to the guidelines and protocols formulated by the Institutional Animal Care and Use Committee (IACUC) of the University of Michigan. These guidelines align with the National Institutes of Health (NIH) standards for the care and use of animals in research (Protocol number: PRO00009613, approved on 24 April 2023). We previously described a generation of *Evc2* conditional mutant mouse line [23]. Details of other mutant mouse lines were reported somewhere else [47,48,49,50,51]. These mutant mice were maintained on a mixed genetic background of C57BL6/J and 129S6 strains. Initially, we compared male and female mice’s sexual dimorphisms, then we focused on female mice for further quantifications.

### 4.2. TMJ Cell Harvest and Culture

Primary TMJ cells were isolated from 8–12-week-old mice. A ~2.0 mm wide tissue section, including the condyle cartilage layer, the ligament attaching sites, and TMJ disc, was dissected using sterile technique. TMJ tissues were transferred into a 15 mL tube containing PBS, then PBS was aspirated and replaced with dissociation buffer (2 mg/mL collagenase and 0.3 mg/mL dispase II in MEM-Alpha, MilliporeSigma, Burlington, MA, USA). Samples were incubated for 2 h at 37 °C with gentle agitation. Following incubation, the suspensions were filtered through a 70 μm strainer, rinsed with 5 mL cold medium, and centrifuged at 1200 rpm for 5 min at room temperature. Supernatant was aspirated, and cells were washed in 1× phosphate buffered saline (PBS, MilliporeSigma, Burlington, MA, USA), followed by another centrifugation at 1200 rpm for 5 min at 4 °C. Cells were re-suspended in basal medium. The basal medium for the CFU-F assay was prepared according to previous publications [52]: MEM-Alpha (Invitrogen, Carlsbad, CA, USA) with 20% FBS, Glutamax (Invitrogen, Carlsbad, CA, USA), 1% penicillin/streptomycin, and 100 μM 2-mercaptoethanol (Gibco, Waltham, MS, USA).

### 4.3. Cell Counting and Preparation

After cell isolation, total nucleated and viable cell counts were determined using a hemocytometer (Fisher, Waltham, MS, USA) with 3% acetic acid and methylene blue to identify nucleated cells, and trypan blue staining for viability assessment (MilliporeSigma, Burlington, MA, USA). Cells were plated at a density of 1 × 10^5^ cells per 35 mm dish. The first medium change was performed four days after initial incubation, and subsequently, the medium was changed every other day.

### 4.4. Colony Formation Unit Fibroblast (CFU-F) Assay

CFU-F assays are employed to evaluate the proliferative potential of progenitor cell populations. We chose the CFU-F assay because it is a robust and quantitative method for assessing the ability of isolated TMJ-derived cells to proliferate and to form colonies, which is indicative of stem cell activity and self-renewal capacity. After 15 days of incubation, the culture dishes were carefully examined for colony formation using a light microscope (Olympus, Tokyo, Japan). Individual colonies were identified as discrete clusters of adherent cells, clearly distinguishable from surrounding single cells or smaller aggregates. For consistency, a colony-forming unit was defined as a cell cluster comprising more than 60 cells. This threshold was set to ensure that only robustly proliferative groups of progenitor cells were included in the analysis, thereby minimizing the potential for counting non-clonal or loosely associated cell groupings. Colonies meeting this criterion were systematically counted across each dish, and the total number of qualifying colonies was recorded for quantitative assessment and statistical analysis. Colonies were stained with crystal violet and visualized by light microscopy for quantitative analysis. Alternatively, when cells were isolated from *Gli1Cre^ERT^*; *R26R^TdTomato^* mice, TdTomato-positivity was identified by red fluorescence, and images were acquired using a confocal microscope (Nikon, Tokyo, Japan).

### 4.5. Osteogenic Differentiation Protocol

After 15 days of CFU-F, colonies were cultured in Dulbecco’s α-MEM medium supplemented with 20% fetal bovine serum (FBS, Biowest USA, Lakewood Ranch, FL, USA) supplemented with 100 nM dexamethasone, 10 mM β-glycerophosphate (MilliporeSigma, Burlington, MA, USA), and 50 µg/mL ascorbic acid (MilliporeSigma, Burlington, MA, USA) to induce osteogenic differentiation. The induction medium was refreshed every 2–3 days throughout the differentiation period, which lasted 3, 4, and 5 weeks. Following induction, osteogenic differentiation was evaluated by Alizarin Red S staining to visualize calcium deposition.

### 4.6. Chondrogenic Differentiation Protocol

Chondrogenic differentiation was performed using the StemPro™ Chondrogenesis Differentiation Kit (Thermo Fisher Scientific, Waltham, MA, USA) according to the manufacturer’s instructions. After 15 days of CFU-F, colonies were trypsinized and seeded at a density of 2.5 × 10^5^ cells per tube to form micromass cultures. The cell pellets were cultured in chondrogenic differentiation medium provided by the kit, with medium changes every 2–3 days, for a total induction period of 3 and 4 weeks. After differentiation, chondrogenesis was evaluated by staining with Alcian Blue to detect sulfated glycosaminoglycans.

### 4.7. Fluorescence Histology Analysis

Samples were embedded in optimal cutting temperature (OCT) compound and cryosectioned. Cryosections were incubated for 1 h in PBS containing 0.1% Triton X-100 (MilliporeSigma, Burlington, MA, USA) and then mounted with DAPI-containing mounting medium (Thermo Fisher Scientific, Waltham, MA, USA) and covered with coverslips. Immunofluorescent imaging was performed using a Leica Thunder imaging system (Leica Microsystems, Wetzlar, Germany), with image overlays created using the microscope’s integrated software (Leica Application Suite, LAS X M205 FA/M205 FCA) to visualize brightfield, DAPI, and fluorescence signals.

### 4.8. Label-Retention Assay for Slow-Cycling Cells

To identify and track slow-cycling progenitor cells within the TMJ enthesis, we utilized *Gli1Cre^ERT^*; *rTta*; *TetO-H2B-GFP* triple transgenic mice, which enable inducible expression of a histone H2B-GFP label under Tet-ON control in *Gli1*-expressing cells. Mice were administered tamoxifen (5 mg/kg body weight, intraperitoneally, MilliporeSigma, Burlington, MA, USA) to induce Cre-mediated recombination specifically in *Gli1^+^* cells. Concurrently, doxycycline chow was provided for seven days to activate reverse tetracycline transactivator (rTta)-dependent transcription and drive expression of H2B-GFP in targeted cells.

After the labeling period, doxycycline was withdrawn, allowing for the gradual dilution of H2B-GFP signal due to cell division; thus, cells retaining bright H2B-GFP fluorescence over subsequent chase periods were identified as slow-cycling, label-retaining cells. Tissue samples were harvested 4 weeks post-labeling and processed for cryosectioning. Retention of the H2B-GFP label was assessed using fluorescence microscopy (Leica Thunder imaging system), and labeled cells were characterized in the TMJ enthesis regions.

### 4.9. Quantitative RT-PCR

The TMJ tissues were carefully dissected for quantitative reverse transcription PCR (qRT-PCR) analysis to determine relative gene expression levels. RNA was extracted from each tissue sample, reverse transcribed into cDNA, and amplified using specific primers (see Table S1 for primer sequences). Target genes included *Gli1*, *Yap1*, *Wwtr1*, and *Piezo1* with *Gapdh* serving as the reference (housekeeping) gene.

For each sample, cycle threshold (Ct) values were obtained for both target and reference genes. Gene expression was normalized within each sample using the difference in Ct values (ΔCt = Ct_target − Ct_reference). Relative gene expression (fold change) was subsequently calculated using the 2^(−ΔCt)^ method.

### 4.10. Statistical Analysis

Data normality was evaluated using the Shapiro–Wilk test. For datasets with confirmed normal distribution, Student’s *t*-test was employed for group comparisons. If normality was not established, the Wilcoxon test was used to determine the significance of differences between two unpaired groups (two-tailed analysis). Statistical significance was denoted as follows: *p* < 0.05 (*), *p* < 0.01 (**), *p* < 0.001 (***), and *p* < 0.0001 (****). For other analyses involving multiple group comparisons, ordinary one-way ANOVA was performed to assess statistical significance.

## 5. Conclusions

Our study firmly establishes the TMJ ligament attachment sites as a distinct niche for *Gli1^+^* progenitor-like cells with robust CFU-F capacity and multipotent differentiation potential. Through region-specific dissection and functional assays, we demonstrate that these cells are slow cycling, display unique morphological features, and are responsive to Hedgehog signaling. Disruption of Evc2 specifically in *Gli1^+^* cells lead to paradoxical upregulation of Gli1 and mechanosensory genes, and results in enhanced colony formation, demonstrating a novel regulatory mechanism linking Evc2/Hedgehog signaling to TMJ progenitor cell behavior. This broadens our understanding of niche integration, regulatory logic, and compartmental interdependency in joint progenitor cell biology. By elucidating new mechanisms of Hedgehog and mechanosensory crosstalk and revealing the functional collaboration between ligament- and cartilage-derived progenitor cells, we provide insights that are relevant not only for the TMJ but for the fundamental regulation of stem/progenitor cell dynamics in joint tissues. Collectively, these findings provide new insights into the cellular hierarchy and regulatory landscape of TMJ ligament-derived progenitors, advancing our understanding of TMJ biology and the cellular dynamics underlying joint maintenance. These findings lay the groundwork for future research targeting a detailed characterization of this stem cell population to enhance TMJ repair and to prevent degenerative joint disease. Ultimately, understanding the molecular mechanisms governing these progenitor cells may open new avenues for therapeutic interventions in TMJ disorders.

## Data Availability

The original contributions presented in this study are included in the article/Supplementary Materials. Further inquiries can be directed to the corresponding author.

## Supplementary Materials

The following supporting information can be downloaded at: https://www.mdpi.com/article/10.3390/ijms27073324/s1.

## Abbreviations

The following abbreviations are used in this manuscript:

TMJ	temporomandibular joint
TACs	transit amplifying cells
TMD	temporomandibular disorders
HH	Hedgehog
CFU-F	colony-forming unit fibroblast
cKO	conditional knockout
FBS	fetal bovine serum
OCT	optimal cutting temperature
rTta	reverse tetracycline transactivator
qRT-PCR	quantitative reverse transcription PCR
OA	Osteoarthritis
FCSC	fibrocartilage stem cells
