# Supplementary material for: Gli1+ Cells Exhibit Clonogenicity and Slow-Cycling Features at the Temporomandibular Joint (TMJ) Enthesis–Condyle Interface
Source: Int J Mol Sci. 2026 Apr 7;27(7):3324. doi: 10.3390/ijms27073324 (PMC13073872; doi:10.3390/ijms27073324)
Supplement: Supplementary file 1 [file ijms-27-03324-s001.zip › ijms-4077086-supplementary.pdf]

Table S1: Quantitative Real Time PCR (qRT-PCR) Primers;

Mouse GAPDH Forward	AGGTCGGTGTGAACGGATTTG
Mouse GAPDH Reverse	AGGTCGGTGTGAACGGATTTG
Mouse Gli1 Forward	CCAAGCCAACTTTATGTCAGGG
Mouse Gli1 Reverse	AGCCCGCTTCTTTGTTAATTTGA
Mouse Yap1 Forward	GACTCCGAATGCAGTGTCTTC
Mouse Yap1 Reverse	TGTTGAGGAAGTCGTCTGGG
Mouse Wwtr1 Forward	TGCTACAGTGTCCCCACAAC
Mouse Wwtr1 Reverse	TGACCGGAATTTTCACCTGT
Mouse Piezo 1 Forward	TCATCATCCTTAACCACATGGTG
Mouse Piezo 1 Reverse	TGAAGACGATAGCTGTCATCCA
